# The Association Between Total Protein Intake and All-Cause Mortality in Middle Aged and Older Korean Adults With Chronic Kidney Disease

**DOI:** 10.3389/fnut.2022.850109

**Published:** 2022-04-04

**Authors:** Yu-Jin Kwon, Hye Sun Lee, Go Eun Park, Hyung-Mi Kim, Jung Joo Lee, Woo Jeong Kim, Ji-Won Lee

**Affiliations:** ^1^Department of Family Medicine, Yongin Severance Hospital, Yonsei University College of Medicine, Seoul, South Korea; ^2^Biostatistics Collaboration Unit, Department of Research Affairs, Yonsei University College of Medicine, Seoul, South Korea; ^3^Department of Food and Nutrition, Dongduck Women’s University, Seoul, South Korea; ^4^Nutrition Team, Yongin Severance Hospital, Yongin, South Korea; ^5^Department of Nutrition Service, Gangnam Severance Hospital, Seoul, South Korea; ^6^Department of Family Medicine, Severance Hospital, Yonsei University College of Medicine, Seoul, South Korea

**Keywords:** protein intake, chronic kidney disease, mortality, Korean Genome and Epidemiology Study, nutrition

## Abstract

**Background and Aims:**

Over the past decades, the optimum protein intake for patients with chronic kidney disease (CKD) has been an important, controversial issue. Dietary protein restriction has been commonly recommended for patients with CKD for preserving kidney function. However, evidence of the associations between long-term protein intake and mortality is not consistent in patients with CKD. Therefore, we aimed to examine the associations between total protein intake and all-cause mortality in Korean adults with CKD.

**Methods:**

From three sub-cohorts of the Korean Genome and Epidemiology Study (KoGES) starting from 2001, total 3,892 participants with eGFR < 60 mL/min/1.73 m^2^ (CKD stage 3–5) were included in this study. Dietary data were collected using food-frequency questionnaires at baseline. Deaths were followed from 2001 to 2019. Cox proportional hazards regression model was used to evaluate the association between protein intake and all-cause mortality.

**Results:**

During a median follow-up (min-max) of 11.1 years (0.3–15.1), 602 deaths due to all causes of mortality were documented. After adjustment for covariates, higher total protein intake was not associated with all-cause mortality [highest vs. lowest quintile of total protein intake (g/kg/day) and proportion (%) (Q5 vs. Q1), HR = 1.14 (0.75–1.72), and HR = 0.87 (0.67–1.13)] in CKD stage 3–5 patients.

**Conclusion:**

Dietary protein intake was not associated with mortality from all causes in patients with CKD. Further research is needed to establish optimal protein intake levels and examine the impact of the dietary source of protein on various health outcomes and mortality in CKD.

## Introduction

Chronic kidney disease (CKD) is a progressive condition characterized by a gradual loss of kidney function, causing kidney damage over time (1). Over the past 27 years, the burden of CKD has not lessened to the same extent as other non-communicable diseases, and its prevalence was estimated to be approximately 10–15% globally (2). In Korea, the prevalence of CKD in adults aged 20 years and older was estimated to be 8.2% (3). CKD is associated with poor health-related quality of life, premature death, and a twofold increase in cardiovascular disease (CVD) prevalence, causing around 1.2 million deaths in 2017 (2, 4).

Although genetic factors play an important role in the incidence and progression of CKD, most cases are attributed to nutritional factors and are largely preventable (5). Therefore, dietary modifications and maintaining adequate nutrient homeostasis should be a fundamental strategy in treating patients with CKD (6). For several decades, low protein intake has been recommended for patients with CKD to slow its progression to end-stage kidney disease and delay renal replacement therapy (7–10). This recommendation is based on the metabolic effects of low protein intake, such as lowering intraglomerular pressure, uremic toxin, and proteinuria, along with improving phosphocalcic metabolism and insulin resistance (11, 12).

In recent years, several clinical studies and meta-analyses have proved the effectiveness of a low protein diet (LPD) on kidney outcomes (11, 13–15). This evidence was included in the 2020 version of the Kidney Disease Outcomes Quality Initiative guidelines, wherein the recommended protein intake for adult patients with metabolically stable, non-diabetic CKD and diabetic CKD, CKD stage 5 on maintenance hemodialysis or peritoneal dialysis was 0.55–0.60, 0.6–0.8, and 1.0–1.2 g/kg/day, respectively (7).

However, despite the available evidence on the benefits of protein restriction, the renoprotective effect of LPD has been debatable due to conflicting results (16, 17). Moreover, there is not enough data supporting the role of an LPD in reducing mortality.

There are substantial differences in dietary habits, food selections, and cooking methods between various populations. The typical American diet contains approximately twice the protein intake recommended by US dietary guidelines (18). According to the 2001–2014 National Health and Nutrition Examination Survey in the United States, the average protein intake in the older adults aged ≥ 71 y was 1.00 g/kg/day in women and 1.11 g/kg/day in men (19). Whereas, more than half of the Korean older adults ≥ 60 years consume less protein than the Korean recommended daily allowance of 0.91 g/kg (20). In the American and European study populations, the major animal-based protein sources were red and processed meat, whereas in the Japanese populations, animal-based protein intake was lower, and the main source of protein was fish (21–23). The quantity and quality of protein intake based on race and ethnicity can affect various health outcomes, including CKD progression, quality of life, morbidity, and mortality (1). To date, there have been few studies investigating the association between protein intake and mortality and patients with CKD, especially in the Asian population. Therefore, we aimed to comprehensively evaluate the association between protein intake and all-cause mortality in adult Korean patients with CKD, using large-scale cohort data.

## Materials and Methods

### Study Population

This study used data from population-based cohorts in the Korean Genome and Epidemiology Study (KoGES), including the KoGES_Ansan and Ansung study (community-based cohort in urban and rural counties), the KoGES_health examinee study (HEXA study) (national health examinee registry), and the KoGES_cardiovascular disease association study (CAVAS study) (community-based cohort in rural counties). In the KoGES, men and women aged over 40 years were recruited for the baseline survey. In the KoGES_Ansan and Ansung study, a total of 10,030 participants aged 40-69 years were voluntarily enrolled at baseline between 2001 and 2002. In the KoGES_HEXA, a total of 173,357 participants were enrolled at baseline from 2004 to 2013. In the KoGES_ CAVAS, a total of 28,338 participants were enrolled at baseline from 2005 to 2011. Detailed information about KoGES were provided in the following website and a previous study (24).^1^

During the recruitment process, participants were asked to volunteer through on-site invitations, mailed letters, telephone calls, media campaigns, or community leader-mediated conferences. A total of 211,571 participants were enrolled at baseline in the KoGES survey. We excluded participants with missing data related to lifestyle (*n* = 2,231), laboratory tests (*n* = 5,853), and nutritional intake (*n* = 14,007), along with implausible daily total calorie intake (< 500 kcal or > 6,000 kcal) who report energy intake levels at the low and high ends of the spectrum (25). After excluding 54,530 participants without mortality data and 63 participants who died within the year of enrollment, a total of 143,050 participants were selected (Supplementary Figure 1). Finally, a total of 3,892 participants with an estimated glomerular filtration rate (eGFR) of < 60 mL/min/1.73 m^2^ were included in the analysis. Among the 3,892 participants, 602 deaths were documented during the follow-up time. This study was approved by the institutional review boards of Yongin Severance Hospital and written informed consents were obtained from all participants.

### Covariates

All participants underwent a health-related survey, and their baseline anthropometric measurements were collected at the first visit. Anthropometric measurements were performed using validated and standardized protocols. Body mass index (BMI) was calculated as body weight (kg) divided by square of height (m). Waist circumference was measured using the measuring tape at the midline level between the inferior margin of the ribs and the superior border of the iliac crest. Smoking status was categorized into current smokers, former smokers, and non-smokers. A current smoker was defined as any participant who currently smoked and has smoked at least 100 cigarettes during their lifetime. Former smokers were defined as adults who smoked at least 100 cigarettes in their lifetime and had quit smoking. Never smokers were defined as adults who had never smoked or smoked < 100 cigarettes in their lifetime. The status of alcohol intake was categorized into current drinker, former drinker, and non-drinker. A regular exerciser was defined as a person who regularly engaged in exercise that is enough to sweat more than once a week. Hypertension was defined as a systolic blood pressure of ≥ 140 mmHg, diastolic blood pressure of ≥ 90 mmHg, or those taking antihypertensive medications. Diabetes was defined as a glycated hemoglobin level of ≥ 6.5%, fasting plasma glucose concentration of ≥ 126 mg/dL after a ≥ 8 h fast, or those taking anti-diabetic medications. Dyslipidemia was defined as a total cholesterol level of ≥ 200 mg/dL, triglyceride ≥ 150 mg/dL, or those taking anti-dyslipidemia medications.

Blood samples were obtained after > 8 h of fasting and were analyzed in a central laboratory. The following biochemical data were determined: concentrations of creatinine, blood urea nitrogen, glucose, glycated hemoglobin, total cholesterol, high-density lipoprotein cholesterol, low-density lipoprotein cholesterol, triglyceride, alanine aminotransferase, and aspartate aminotransferase. eGFR was calculated using the Modification of Diet in Renal Disease Study equation (26). CKD was defined as an eGFR of < 60 mL/min/1.73 m^2^ (27).


GFR(mL/min/1.73m)2=175×(S)cr×-1.154(Age)×-0.203(0.742iffemale)×(1.212ifAfricanAmerican).

### Mortality Ascertainment

The mortality status was followed by data linkage with national data sources based on the unique personal identification key code system. The KoGES data have been linked to national data sources (Korea National Statistical Office), including death records, to evaluate mortality status. Participant deaths were tracked from January 2001 until December 2019. The underlying causes of death were based on the Korean Standard Classification of Diseases codes listed in the National Death Index. All-cause mortality was defined as all deaths of specified and unknown causes.

### Dietary Assessment

Information on nutritional intake was derived from the FFQ data. In 2001, a semi-quantitative food frequency questionnaire (FFQ) containing 103 food items was developed for the KoGES survey (28). Each questionnaire evaluated how often the participants consumed each food (never or almost never, once a month, 2–3 times per month, 1–2 times per week, 3–4 times per week, 5–6 times per week, once a day, twice per day, or three times per day) and how much they consumed during each session (1/2 serving, 1 serving, or 2 or more servings) over the past 1 year.

Detailed information regarding the protocol and results of a validation study for the FFQ have been described in the previous studies (24, 28). After conducting the survey using the 1st edition FFQ in 2001, a revised version of the questionnaire including 106 items was released in 2004. Therefore, the KoGES-Ansan-Ansung baseline study contains FFQ data including 103 food items, while the KoGES-HEXA and KoGES_CAVAS contain FFQ data including 106 food items. The integrated three KoGES datasets provided the daily intake amounts of only 23 nutrients using the processed data. Carbohydrate intake, fat intake, and protein intake were recorded as g/day. The proportions of carbohydrate, protein, and fat intake were calculated as carbohydrate intake (g/day) or protein intake (g/day) × 4 kcal/total energy intake (kcal/day) × 100, and fat intake (g/day) × 9 kcal/total energy intake (kcal/day) × 100. Protein intake per kg per body weight was also recorded. Protein intake (g/kg/day) and the proportion of protein intake were classified in quintiles.

### Statistical Analysis

Data were analyzed from 2001 to 2013. Participant deaths were tracked until December 2019. The follow-up period for each study participant was calculated as the time from their KoGES initial assessment to either an event of mortality or the date of death. Data were presented as mean ± standard deviation (SD) for continuous variables or number (%) for categorical variables. Protein intake (g/kg/day) was divided into quintiles. Baseline characteristics of the study population according to the protein intake quintiles were compared using the one-way analysis of variance (ANOVA) for continuous variables and the Chi-squared test for categorical variables. A Cox proportional hazard spline curve was used to assess the association between dietary protein intake and all-cause mortality risk. Warranty period was defined as the time to reach the cumulative mortality rate of > 1% for each group. Person-time is an estimate of the actual time-at-risk in years. Incidence per 1,000 person-years was calculated for each group. Cox proportional hazards regression model was used to evaluate the association between protein intake and all-cause mortality. We adjusted for covariates in 3 models. Model 1 was adjusted for age, sex, and BMI. Model 2 was adjusted for age, sex, BMI, smoking, alcohol intake, exercise, and total calorie intake (for the isocaloric model). While model 3 was further adjusted for hypertension, dyslipidemia, and diabetes. We performed sensitive analysis to investigate the protein intake and mortality in CKD patients according to presence of diabetes and total calorie intake 20 kcal/kg/day criteria.

We used SAS version 9.4 (SAS Institute, Cary, NC, United States) and R package version 4.0.3.^2^ All statistical tests were two-sided, and *P* < 0.05 was considered statistically significant.

## Results

Baseline demographics, lifestyle, and nutrient intake were organized according to quantiles of total protein intake (g/kg/day) and are presented in Table 1. Of the 3,892 patients with CKD, 1,495 (38.4%) were men, and the mean (SD) age was 62.9 ± 8.3 years. The proportion of protein intake of the population was 12.64 ± 2.53%, and the mean protein intake was 0.82 ± 0.35 g/kg/day. During a median follow-up (min–max) of 11.08 (0.33–15.08) years, we documented 602 deaths due to all causes (Supplementary Table 1). Participants with the highest intake of protein were more likely to be young (*P* < 0.001), with a lower BMI, waist circumference (*P* < 0.001), and triglyceride levels (*P* < 0.001). They were more likely to drink alcohol and exercise regularly (*P* < 0.001) and were more likely to live in urban areas (*P* < 0.001), as compared to the lower protein intake group. There were no significant differences in blood pressure and glucose levels, glycated hemoglobin, total cholesterol, and LDL cholesterol. Regarding underlying disease, the proportion of patients with dyslipidemia was significantly higher in the lowest protein intake group than the highest protein intake group. The proportion of those with hypertension and diabetes was not significantly different between the two groups. Regarding nutritional intake, total calorie intake, carbohydrate intake (g/day), and fat intake (g/day and %) were significantly higher in participants with a higher protein intake than in those with lower protein intake. The proportion of carbohydrate intake (%) was significantly lower in patients with CKD with higher protein intake than in those with lower protein intake. The results from the *post hoc* analysis between the quintiles based on dietary protein intake are shown in Supplementary Table 2.

**TABLE 1:** Baseline characteristics of the cohort according to protein intake (g/kg/day).

	Q1 (0.179, 0.546)	Q2 (0.546, 0.683)	Q3 (0.682, 0.835)	Q4 (0.836, 1.041)	Q5 (1.041, 3.573)
N	778	779	778	779	778
Sex (men), *n* (%)	271 (34.8)	296 (38.0)	312 (40.1)	330 (42.4)	286(36.8)
Age, years	65.2 ± 8.0	63.0 ± 8.4	62.8 ± 8.2	62.3 ± 8.0	60.9 ± 8.2
BMI, kg/m^2^	25.9 ± 3.4	25.1 ± 2.9	24.9 ± 3.0	24.4 ± 2.9	23.7 ± 2.7
WC, cm	87.8 ± 9.0	86.1 ± 8.6	85.5 ± 8.7	84.3 ± 8.4	81.9 ± 8.5
SBP, mmHg	128.6 ± 18.2	128.0 ± 18.0	127.6 ± 16.2	127.2 ± 17.7	126.5 ± 17.5
DBP, mmHg	77.9 ± 10.2	77.9 ± 10.5	77.8 ± 10.0	77.2 ± 10.5	77.3 ± 10.6
FBG, mg/dl	104.7 ± 31.1	104.9 ± 39.1	105.0 ± 34.1	103.7 ± 38.5	102.0 ± 34.2
HbA1c,%	6.24 ± 1.05	6.28 ± 1.19	6.15 ± 1.27	6.13 ± 1.32	6.08 ± 0.96
TC, mg/dl	196.2 ± 40.8	196.7 ± 40.0	198.7 ± 41.2	198.1 ± 37.6	198.1 ± 39.5
HDL-C, mg/dl	44.2 ± 11.6	44.9 ± 11.4	46.0 ± 11.9	47.2 ± 12.2	48.8 ± 12.3
LDL-C, mg/dl	119.5 ± 37.6	120.7 ± 35.8	120.2 ± 37.0	121.1 ± 33.9	121.4 ± 35.4
TG, mg/dl	164.0 ± 98.3	157.0 ± 94.4	162.6 ± 105.6	150.0 ± 86.6	140.9 ± 86.6
BUN, mg/dl	20.6 ± 9.3	20.6 ± 10.5	19.6 ± 6.5	20.4 ± 7.8	20.4 ± 8.3
Cr, mg/dl	1.37 ± 0.93	1.38 ± 0.81	1.31 ± 0.61	1.34 ± 0.61	1.38 ± 1.06
eGFR, mL/min/1.73 m^2^	51.4 ± 10.0	51.7 ± 10.2	52.7 ± 8.6	52.4 ± 9.3	52.7 ± 9.9
AST, IU/L	25.8 ± 9.4	26.3 ± 11.3	25.3 ± 10.	24.7 ± 8.6	25.4 ± 11.5
ALT, IU/L	23.0 ± 13.2	23.8 ± 14.7	22.5 ± 11.5	22.7 ± 12.9	22.6 ± 14.9
**Smoking status, *n* (%)**					
Never smoker	561 (72.1)	548 (70.4)	545 (70.1)	524 (67.3)	561 (72.1)
Former smoker	148 (19.0)	166 (21.3)	158 (20.3)	171 (22.0)	145 (18.6)
Current smoker	69 (8.9)	65 (8.3)	75 (9.6)	84 (10.8)	72 (9.3)
**Alcohol intake, *n* (%)**					
Never drinker	491 (63.1)	487 (62.5)	461 (59.3)	440 (56.5)	442 (56.8)
Former drinker	87 (11.2)	67 (8.6)	64 (8.2)	50 (6.4)	44 (5.7)
Current drinker	200 (25.7)	225 (28.9)	253 (32.5)	289 (37.1)	292 (37.5)
Regular exercise, *n* (%)	260 (33.4)	337 (43.3)	347 (44.6)	395 (50.7)	408 (52.4)
HTN, *n* (%)	228 (29.3)	213 (27.3)	215 (27.6)	216 (27.7)	200 (25.7)
DM, n (%)	137 (17.6)	139 (17.8)	131 (16.8)	110 (14.1)	119 (15.3)
Dyslipidemia, *n* (%)	515 (66.2)	488 (62.6)	508 (65.3)	483 (62.0)	461 (59.3)
**Residential area**					
Urban, *n* (%)	335 (43.1)	450 (57.8)	475 (61.1)	527 (67.7)	590 (75.8)
Rural, *n* (%)	443 (56.9)	329 (42.2)	303 (39.0)	252 (32.4)	188 (24.2)
Total energy intake, kcal/day	1,072.3 ± 276.5	1,371.3 ± 257.3	1,555.6 ± 274.6	1,757.6 ± 300.8	2,164.9 ± 507.9
CHO (g/day)	213.1 ± 59.3	265.2 ± 56.3	292.4 ± 58.9	320.1 ± 64.1	367.7 ± 87.5
CHO (%)	79.2 ± 5.0	77.1 ± 5.1	74.9 ± 5.2	72. ± 5.5	68.1 ± 6.7
Fat, g/day	9.3 ± 4.9	14.4 ± 6.0	19.3 ± 7.5	25.2 ± 9.1	40.0 ± 18.4
Fat,%	8.1 ± 4.2	9.6 ± 4.2	11.3 ± 4.2	13.0 ± 4.4	16.4 ± 5.2
Protein, g/day	28.5 ± 7.0	39.3 ± 7.1	47.7 ± 8.0	57.8 ± 9.7	80.9 ± 23.3
Protein,%	10.8 ± 1.8	11.6 ± 1.9	12.4 ± 1.9	13.3 ± 2.1	15.0 ± 2.5

*BMI, body mass index; WC, waist circumference; SBP, systolic blood pressure; DBP, diastolic blood pressure; FBG, fasting blood glucose; HbA1c, glycated hemoglobin; TC, total cholesterol; HDL-C, high-density lipoprotein cholesterol; LDL-C, low-density lipoprotein cholesterol; TG, triglyceride; BUN, blood urea nitrogen; Cr, creatinine; eGFR, estimated glomerular filtration rate; AST, aspartate transaminase; ALT, alanine transaminase; HTN, hypertension; DM, diabetes; CHO, carbohydrate.*

Table 2 shows the warranty periods and incidences per 1,000 person-years [95% confidence interval (CI)] for all-cause mortality according to protein (g) per kilogram of body weight and the proportion of protein intake. Regarding protein intake (g/kg/day), the warranty periods were the longest in Q2 and Q5. Regarding protein intake proportion (%), the warranty period was longest in Q5. Incidence per 1,000 person-years (95% CI) was lower in the highest quintile protein intake (g/kg/day) group, in those who consume protein over 1.2 g/kg/day, and in the highest quintile of protein intake proportion (%).

**TABLE 2:** Warranty periods for all-cause mortality according to protein intake (g/kg/day).

Variables	Warranty Period	*n*	Person-Time	Events,	Incidence Per 1000 Person-Years
	(1%)		(Years)	*n* (%)	(95% CI)
**Protein intake (g/kg/day)**					
Q1 (0.179, 0.546)	1.666	778	8,122.6	157 (20.2)	19.3 (9.7–29.0)
Q2 (0.546, 0.683)	3.334	779	8,219.5	120 (15.5)	14.6 (6.2–23.0)
Q3 (0.683, 0.835)	1.748	778	8,199.5	113 (14.6)	13.8 (5.6–22.0)
Q4 (0.836, 1.040)	1.504	779	8,098.9	117 (15.1)	14.4 (6.1–22.8)
Q5 (1.041, 3.573)	3.334	778	8,244.7	95 (12.3)	11.5 (4.0–19.0)
**Protein intake (g/kg/day)**					
< 0.8	1.916	2,181	22,925.2	368 (16.9)	16.1 (10.8–21.3)
0.8–1.2	2.165	1,249	13,009.1	187 (15)	14.4 (7.8–21.0)
> 1.2	3.331	462	4,951.0	47 (10.2)	9.5 (0.7–18.3)
**Protein (%)**					
Q1 (6.9, 10.5)	1.666	778	8,178.3	174 (22.4)	21.3 (11.1–31.4)
Q2 (10.5, 11.7)	1.748	779	8,263.0	115 (14.8)	13.9 (5.7–22.1)
Q3 (11.7, 12.9)	2.499	778	8,185.1	107 (13.8)	13.1 (5.1–21.1)
Q4 (12.9, 14.6)	1.833	779	8,059.4	109 (14)	13.5 (5.4–21.6)
Q5 (14.6, 25.5)	3.329	778	8,199.4	97 (12.5)	11.8 (4.2–19.4)

**FIGURE 1:**
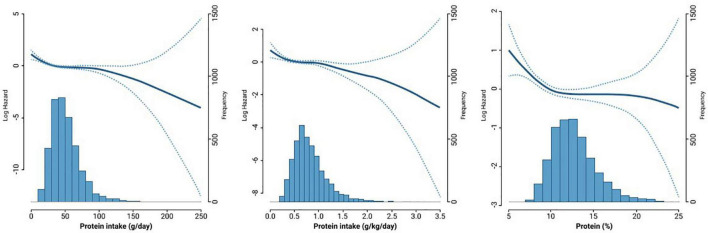
The density plot of protein intake and Cox proportional hazard spline curve showing the association between protein intake and all-cause mortality.

Figure 1 shows the density plot of protein intake and Cox proportional hazard spline curve, which depicts the association between protein intake and all-cause mortality. In most protein intake ranges, there were non-linear associations between protein intake and all-cause mortality.

Table 3 shows the results from multiple Cox proportional hazard regression analyses for all-cause mortality according to protein intake. Higher protein intake (g/kg/day) was not associated with all-cause mortality [Q5 vs. Q1, HR, 1.14 (95% CI, 0.75–1.72) in model 3]. We additionally divided protein intake between three groups based on the recent Kidney Disease: Improving Global Outcome guidelines (<0.8, 0.8–1.2, and >1.2 g/kg/day). (29) While comparing protein intake of >1.2 g/kg/day or 0.8–1.2 g/kg/day and <0.8 g/kg/day, higher protein intake was not associated with the risk of all-cause mortality [>1.2 g/kg/day vs. <0.8 g/kg/day, HR, 1.06 [95% CI, 0.71–1.57 in model 3; 0.8–1.2 g/kg/day vs. <0.8 g/kg/day, HR, 1.14 (95% CI, 0.91–1.41) in model 3]. Regarding the proportion of protein intake, compared to Q1, the HRs and 95% CIs were 0.85 (0.67–1.08) in Q2, 0.81 (0.63–1.04) in Q3, 0.94 (0.73–1.20) in Q4, and 0.87 (0.67–1.13) in Q5 after adjusting for age, sex, BMI, smoking, alcohol intake, exercise, total calorie, hypertension, diabetes, and dyslipidemia.

**TABLE 3:** Multiple Cox proportional hazard regression analysis for all-cause mortality of protein intake quintile.

	Model 1		Model 2		Model 3	
Variables	HR (95% CI)	*p*-value	HR (95% CI)	*p*-value	HR (95% CI)	*p*-value
**Protein intake (g/kg/day)**						
Q1 (0.179, 0.546)	Ref		Ref		Ref	
Q2 (0.546, 0.683)	0.83 (0.65–1.06)	0.128	0.97 (0.75–1.26)	0.830	0.96 (0.74–1.24)	0.745
Q3 (0.683, 0.835)	0.75 (0.59–0.96)	0.024	0.93 (0.70–1.23)	0.611	0.92 (0.69–1.22)	0.551
Q4 (0.835, 1.041)	0.84 (0.66–1.07)	0.152	1.14 (0.82–1.57)	0.439	1.10 (0.80–1.53)	0.547
Q5 (1.041, 3.573)	0.76 (0.58–0.99)	0.039	1.20 (0.79–1.81)	0.392	1.14 (0.75–1.72)	0.541
**Protein intake (g/kg/day)**						
< 0.8	Ref		Ref		Ref	
0.8–1.2	0.96 (0.80–1.15)	0.632	1.15 (0.92–1.43)	0.212	1.14 (0.91–1.41)	0.255
> 1.2	0.75 (0.55–1.02)	0.064	1.08 (0.72–1.61)	0.707	1.06 (0.71–1.57)	0.795
**Protein intake (%)**						
Q1 (6.9, 10.5)	Ref		Ref		Ref	
Q2 (10.5, 11.7)	0.81 (0.64–1.03)	0.085	0.87 (0.69–1.11)	0.262	0.85 (0.67–1.08)	0.173
Q3 (11.7, 12.9)	0.74 (0.58–0.95)	0.018	0.83 (0.65–1.06)	0.130	0.81 (0.63–1.04)	0.098
Q4 (12.9, 14.6)	0.88 (0.69–1.13)	0.317	0.95 (0.74–1.22)	0.684	0.94 (0.73–1.20)	0.596
Q5 (14.6, 25.5)	0.78 (0.60–1.00)	0.054	0.90 (0.69–1.16)	0.406	0.87 (0.67–1.13)	0.300

*Model 1: adjusted for age, SEX, and BMI.*

*Model 2: adjusted for age, SEX, BMI, smoking, alcohol intake, exercise, and total calorie.*

*Model 3: adjusted for age, SEX, BMI, smoking, alcohol intake, exercise, total calorie, hypertension, diabetes, and dyslipidemia.*

We performed several sensitivity analyses. We examined the association between total protein intake and all-cause mortality in patients with CKD according to the presence of diabetes (Supplementary Table 3). There was no significant association between total protein intake and all-cause mortality in the diabetes and non-diabetes groups. We examined the association between protein intake and mortality in CKD patients according to total calorie intake 20 kcal/kg/day criteria. There was no significant association between total protein intake and all-cause mortality in CKD patients who consume total calorie <20 kcal/kg/day. There was also no significant association between total protein intake and all-cause mortality in CKD patients who consume total calorie over than 20 kcal/kg/day (Supplementary Table 4). We further examined the association between total protein intake and all-cause mortality in patients with CKD according to the CKD stage (e.g., stages 3, 4, and 5). There was also no significant association between total protein intake and all-cause mortality according to CKD stages (data not shown). However, there were very few patients with CKD stages 4 and 5 in the dataset. A small dataset could lead to overfitting problems in logistic regression models.

## Discussion

In this large population cohort of Korean adults with CKD, we found that higher protein intake was not associated with all-cause mortality. Additionally, we observed similar results after stratifying based on CKD stages and the presence of diabetes.

Given the high incidence of CKD and an emergent need for disease management strategies, lifestyle and dietary modifications are cost-effective strategies that may help increase the lifespan and prolong the dialysis-free interval in treating patients with CKD (30). Although consuming adequate dietary protein is critical for maintaining optimal health and various bodily functions (31), patients with CKD have been advised to control their dietary protein intake due to a reduction in glomerular capillary pressure and filtration (32, 33). However, there was no definitive evidence for the optimal level of protein intake, especially when prescribed to patients with various underlying conditions. In the general population, the association between total protein intake and mortality has considerable controversy (23, 34–36). One meta-analysis found that total protein intake was significantly associated with increased mortality risk, while another meta-analysis showed contrary results, stating that total protein intake reduced the risk of all-cause mortality (34, 35). A prospective cohort study conducted in Japan reported a null association between total protein and all-cause mortality (23). Most studies, which analyze the effects of LPD on kidney outcomes have proved the beneficial effects of dietary protein restriction in reducing the decline in renal function and the risk of progression to kidney failure in CKD populations (6, 14, 37). However, unfortunately, the results of a few other studies are not consistent, showing no benefits of protein restriction for patients with CKD (38). In fact, several studies, including the Modification of Diet in Renal Disease study, failed to show the effectiveness of LPD in slowing CKD progression (39). Furthermore, there are only a few studies on the effect of dietary protein intake on mortality in patients with CKD, and the outcomes are majorly inconsistent (34). Recently, in a cohort of adults in the United States of America with impaired kidney function, higher dietary protein consumption was associated with higher mortality rates (40). A prospective, multicenter trial involving 456 patients with CKD showed a borderline significant difference between the low protein diet group and normal protein diet group in terms of renal survival (41). In contrast, several studies commented that dietary protein restriction had no influence on all-cause death in patients with CKD (14, 42). Our results are in line with previous studies indicating a non-significant association between protein intake and mortality.

The underlying mechanism linking dietary protein intake and the mortality risk remain unclear; however, several possible explanations could support our findings. The mean dietary protein consumption in our cohort of patients with CKD is 0.82 (g/kg/day), which remains substantially higher than the recommendations of LPD for patients with CKD; however, it is not clear whether the benefit of protein restriction is warranted in view of the potential for causing malnutrition (14). Under protein restriction, with ≤ 0.6 g/kg/day of protein intake, supplementation with essential amino acids and adequate caloric intake (30–35 kcal/kg/day) are needed to avoid protein-energy wasting and muscle loss, particularly in frail and older CKD patients (43). In addition, usually, a high protein diet, defined as >1.2 g/kg/day, is known to be the threshold to induce significant alterations in renal function and kidney health (44). Notably, recent US cohort studies have revealed that a high dietary protein intake of ≥1.4 g/kg/day was associated with increased mortality, while dietary protein intake levels of 1.0–1.4 and <0.6 g/kg/day were not associated with mortality in participants with impaired kidney function (40). The PROT-AGE group suggested that a protein intake of >0.8 g/kg/day is safe, and GFR monitoring should be performed for patients with CKD with an eGFR of 30–60 mL/min/1.73 m^2^ (45). Therefore, from our study, it is possible that dietary protein consumption may not result in deterioration of renal function. According to the protein leverage hypothesis (46), protein requirements (grams/day) relate to body weight and remain virtually constant across all energy intakes (47, 48). Protein under-consumption leads to an increased intake of carbohydrates (49). Considering that patients with CKD are more likely to die from CVD instead of developing kidney failure (50, 51), excess energy intake from a carbohydrate source could have unfortunate effects on cardiovascular death (52, 53).

While interpreting our findings, protein food sources and the dietary pattern of the Korean diet should be considered. The main characteristic of the Korean diet includes proportionally high consumption of vegetables, moderate-to-high consumption of legumes and fish, and low consumption of red meat (54). According to a previous study using the 1998–2018 Korean Korea National Health and Nutrition Examination Survey Data, Koreans consumed protein from plant sources by 63.07% and animal sources by 36.93% (55). And the primary source of protein intake for Koreans were grains, followed by seafood, meat and legumes (55). Recently, several issues regarding the impact of the dietary source of protein remain a concern. A plant-based protein diet lowers the prevalence of renal function impairment (56–60), and a high intake of legumes, grains, and nuts (major sources of plant proteins) is reportedly associated with a lower risk of all-cause and CVD-related mortality (61, 62). However, red meat intake was strongly associated with ESRD risk in a dose-dependent manner, while other protein sources such as fish, poultry, or dairy products did not show such a deleterious effect (63). Unlike animal protein, plant protein can ameliorate metabolic acidosis in CKD (1, 64) and reduce the generation of uremic toxins, such as p-cresyl sulfate and indoxyl sulfate, which contribute toward CKD progression, CVD, and mortality (65). Also, plant protein is generally low in branched-chain acids and aromatic amino acids (66) and exerts favorable effects on blood pressure and lipid metabolism (60, 64), resulting in decreased risks of CVD (67). Thereby, null associations observed in our study between protein intake and mortality might be explained partly by different dietary patterns and food sources of protein in the Korean population compared with Western dietary patterns. Further prospective and intervention studies with plant-based protein consumption in patients with CKD are needed to address our assumption.

Our study has several limitations. First, the amount of dairy protein intake was assessed using FFQ, which has disadvantages, including inaccuracy of absolute nutrient values and fluctuation of nutrient values. However, FFQ is a validated, relatively cost- and time-effective method to assess nutrient intake and to assess usual and long-term intake in large-scale epidemiologic studies (68), Second, we focused on total protein intake rather than on the type of protein intake. Protein type may be more important for disease progression and mortality than the total amount of protein intake. Third, along with eGFR, urinary albumin/creatinine ratio is important to define CKD. Since we did not obtain information about urinary albumin/creatinine ratio from the KoGES dataset, we defined CKD as eGFR <60 mL/min/1.73 m^2^. Forth, the lack of information on dialysis made it impossible to analyze the effect of dietary protein on mortality by categorizing patients with CKD based on their dialysis requirements. Instead, we conducted sensitivity analyses according to the CKD stages to test the robustness of our results, and there were also no significant associations between total protein intake and all-cause mortality in each stage. However, the KoGES contains very few individuals with advanced CKD; hence further studies specifically targeting advanced patients with CKD and patients on dialysis are needed. Fifth, to clarify the association between dietary total protein intake and mortality in CKD patients, validation is needed in the other larger CKD cohort. Finally, we could not further explore the effect of dietary protein on mortality by specific causes due to the limited number of patients who died. Despite these limitations, this study has several strengths. Our study comprised a prospective design, a large population size, and an extensive, eleven-year follow-up period. Also, to our knowledge, this is the first study to examine the association between total protein intake and mortality risk among adult patients with CKD in an Asian population.

## Conclusion

We could not confirm a causal relationship between dietary protein intake and survival in the Korean population with CKD. Higher protein intake was not associated with all-cause mortality in patients with CKD, and similar results were obtained after stratifying patients based on CKD stages and the presence of diabetes. Based on these findings, our analysis supports that severe protein restriction with less severe kidney failure may not have a clear beneficial effect for all-cause death events. Further research is needed to establish optimal dietary protein intake levels for this population with CKD stage 3–5. Also, additional studies are needed to examine the impact of the dietary source of protein on various health outcomes and mortality.

## Data Availability Statement

The original contributions presented in the study are included in the article/Supplementary Material, further inquiries can be directed to the corresponding author/s.

## Ethics Statement

The studies involving human participants were reviewed and approved by the Yongin Severance Hospital. The patients/participants provided their written informed consent to participate in this study.

## Author Contributions

Y-JK, HL, GP, and J-WL contributed to the conception or design of the work. Y-JK, HL, GP, H-MK, WK, JL and J-WL contributed to the acquisition, analysis, and interpretation of the data, draft the manuscript. All authors critically revised the manuscript, provided final approval and agreed to be accountable for all aspects of the work, ensuring integrity and accuracy.

## Conflict of Interest

The authors declare that the research was conducted in the absence of any commercial or financial relationships that could be construed as a potential conflict of interest.

## Publisher’s Note

All claims expressed in this article are solely those of the authors and do not necessarily represent those of their affiliated organizations, or those of the publisher, the editors and the reviewers. Any product that may be evaluated in this article, or claim that may be made by its manufacturer, is not guaranteed or endorsed by the publisher.

## Footnotes

1
https://www.kdca.go.kr/contents.es?mid=a40504030100

2
http://www.R-project.org

## Supplementary Material

Supplementary Figure 1 Flow chart of the study population.
