# Supplementary material for: The Association Between Total Protein Intake and All-Cause Mortality in Middle Aged and Older Korean Adults With Chronic Kidney Disease
Source: Front Nutr. 2022 Apr 4;9:850109. doi: 10.3389/fnut.2022.850109 (PMC9014017; doi:10.3389/fnut.2022.850109)
Supplement: Supplementary file 1 [file Data_Sheet_1.docx]

**Supplementary materials**

**The association between total protein intake and all-cause mortality in middle aged and older adult with chronic kidney disease**

**Supplementary Figure1.**

**
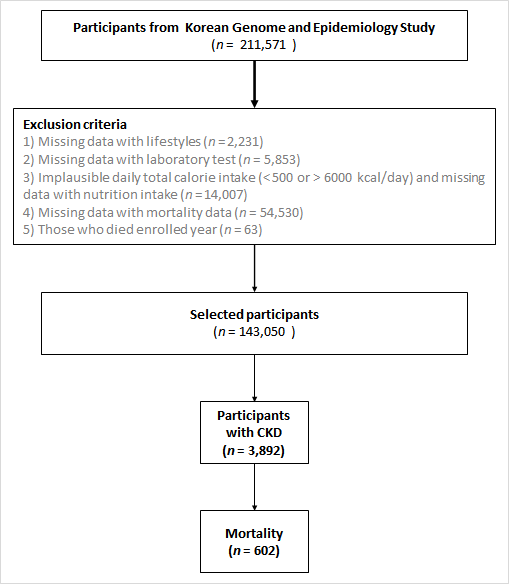
**

**Supplementary Table S1. Baseline characteristics of the cohort according to mortality status**

| **Characteristics** | **Alive** | **All-cause mortality** | **p-value** |
| --- | --- | --- | --- |
| **N** | 3290 | 602 |  |
| **Sex (men), n (%)** | 1209(36.8) | 286(47.5) | <.0001 |
| **Age (years)** | 61.8±8.0 | 68.5±7.3 | <.0001 |
| **BMI (kg/m^2^)** | 24.9±3.0 | 24.2±3.2 | <.0001 |
| **Waist circumference (cm)** | 85.0±8.8 | 85.9±9.3 | 0.0388 |
| **Systolic BP (mmHg)** | 127.1±17.0 | 130.2±19.9 | 0.0005 |
| **Diastolic BP (mmHg)** | 77.8±10.3 | 76.4±10.9 | 0.0026 |
| **Glucose (mg/dl)** | 102.6±33.2 | 112.1±45.7 | <.0001 |
| **HbA1c (%)** | 6.11±1.08 | 6.79±1.71 | <.0001 |
| **Total cholesterol (mg/dl)** | 198.4±39.6 | 193.1±40.8 | 0.003 |
| **HDL-C (mg/dl)** | 46.7±12.0 | 43.4±11.5 | <.0001 |
| **LDL-C (mg/dl)** | 121.1±36.0 | 117.6±35.5 | 0.0272 |
| **Triglycerides (mg/dl)** | 153.9±95.5 | 160.7±91.1 | 0.106 |
| **BUN (mg/dl)** | 20.0±8.2 | 22.3±10.2 | <.0001 |
| **Creatinine(mg/dl)** | 1.3±0.8 | 1.5±1.0 | <.0001 |
| **AST (IU/L)** | 25.3±10.1 | 26.6±11.0 | 0.0177 |
| **ALT (IU/L)** | 23.0±13.6 | 22.6±12.9 | 0.5223 |
| **Smoking status, n (%)** |  |  | <.0001 |
| **Never smoker** | 2378(72.3) | 361(60.0) |  |
| **Former smoker** | 641(19.5) | 147(24.4) |  |
| **Current smoker** | 271(8.2) | 94(15.6) |  |
| **Alcohol intake, n (%)** |  |  | <.0001 |
| **Never drinker** | 1999(60.8) | 322(53.5) |  |
| **Former drinker** | 226(6.9) | 86(14.3) |  |
| **Current drinker** | 1065(32.4) | 194(32.2) |  |
| **Regular exercise, n (%)** | 1542(42.9) | 205(34.1) | <.0001 |
| **Hypertension, n (%)** | 881(26.8) | 191(31.7) | 0.0124 |
| **Diabetes mellitus, n (%)** | 496(15.1) | 140(23.3) | <.0001 |
| **Dyslipidemia, n (%)** | 2083(63.3) | 372(61.8) | 0.4777 |
| **Residential area, n (%)** |  |  | <.0001 |
| **Urban** | 2102(63.9) | 275(45.7) |  |
| **Rural** | 1188(36.1) | 327(54.3) |  |
| **Total energy intake (kcal/day)** | 1603.6±502.9 | 1479.2±458.7 | <.0001 |
| **Carbohydrate intake (g/day)** | 294.6±84.6 | 276.1±79.5 | <.0001 |
| **Carbohydrate (%)** | 74.2±6.7 | 75.4±6.8 | <.0001 |
| **Fat intake (g/day)** | 22.2±15.0 | 18.7±13.2 | <.0001 |
| **Fat (%)** | 11.8±5.3 | 10.8±5.4 | <.0001 |
| **Protein intake (g/day)** | 51.7±22.2 | 45.9±19.4 | <.0001 |
| **Protein (%)** | 12.7±2.5 | 12.2±2.6 | <.0001 |

BMI, body mass index; WC, waist circumference; SBP, systolic blood pressure; DBP, diastolic blood pressure; FBG, fasting blood glucose; TC, total cholesterol; HDL-C, high density lipoprotein cholesterol; LDL-C, low density lipoprotein cholesterol; TG, triglyceride; BUN, blood urea nitrogen; Cr, creatinine; AST, aspartate transaminase; ALT, alanine transaminase; HTN, hypertension; DM, diabetes

**Supplementary Table S2. Post-hoc analysis results from the comparison of study population**

| **Characteristics** | **1 vs 2** | **1 vs 3** | **1 vs 4** | **1 vs 5** | **2 vs 3** | **2 vs 4** | **2 vs 5** | **3 vs 4** | **3 vs 5** | **4 vs 5** |
| --- | --- | --- | --- | --- | --- | --- | --- | --- | --- | --- |
| **N** |  |  |  |  |  |  |  |  |  |  |
| **Sex (men)** | 0.1945 | 0.0318 | 0.0023 | 0.4277 | 0.3945 | 0.0789 | 0.6141 | 0.3652 | 0.1754 | 0.0238 |
| **Age (years)** | <.0001 | <.0001 | <.0001 | <.0001 | 0.6884 | 0.107 | <.0001 | 0.2261 | <.0001 | 0.0006 |
| **BMI (kg/m^2^)** | <.0001 | <.0001 | <.0001 | <.0001 | 0.1863 | <.0001 | <.0001 | 0.001 | <.0001 | <.0001 |
| **Waist circumference (cm)** | 0.0002 | <.0001 | <.0001 | <.0001 | 0.1606 | <.0001 | <.0001 | 0.0045 | <.0001 | <.0001 |
| **Systolic BP (mmHg)** | 0.5275 | 0.231 | 0.1036 | 0.0156 | 0.5711 | 0.3192 | 0.0738 | 0.6676 | 0.2221 | 0.4283 |
| **Diastolic BP (mmHg)** | 0.9521 | 0.9009 | 0.2054 | 0.2642 | 0.8536 | 0.1846 | 0.2392 | 0.2535 | 0.3211 | 0.8812 |
| **Laboratory** |  |  |  |  |  |  |  |  |  |  |
| **Glucose (mg/dl)** | 0.9051 | 0.8624 | 0.563 | 0.1331 | 0.9568 | 0.4853 | 0.1049 | 0.4521 | 0.0939 | 0.3554 |
| **HbA1c (%)** | 0.7206 | 0.4279 | 0.3318 | 0.157 | 0.2295 | 0.1634 | 0.0631 | 0.8607 | 0.5205 | 0.6353 |
| **Total cholesterol (mg/dl)** | 0.825 | 0.2245 | 0.3456 | 0.3596 | 0.3203 | 0.4701 | 0.4868 | 0.7857 | 0.7654 | 0.9787 |
| **HDL-C (mg/dl)** | 0.2606 | 0.0026 | <.0001 | <.0001 | 0.0592 | 0.0001 | <.0001 | 0.0489 | <.0001 | 0.0057 |
| **LDL-C (mg/dl)** | 0.5112 | 0.7096 | 0.369 | 0.3083 | 0.7759 | 0.8091 | 0.717 | 0.5987 | 0.5176 | 0.9036 |
| **Triglycerides (mg/dl)** | 0.1431 | 0.7793 | 0.0036 | <.0001 | 0.2363 | 0.1483 | 0.0008 | 0.0086 | <.0001 | 0.0564 |
| **BUN** | 0.9104 | 0.0357 | 0.7256 | 0.7396 | 0.0269 | 0.6429 | 0.6564 | 0.0801 | 0.0771 | 0.9852 |
| **Creatinine** | 0.8895 | 0.143 | 0.4286 | 0.8947 | 0.1087 | 0.352 | 0.9949 | 0.5005 | 0.1103 | 0.3555 |
| **AST (IU/L)** | 0.2625 | 0.3263 | 0.0422 | 0.4342 | 0.0356 | 0.0016 | 0.0573 | 0.294 | 0.8426 | 0.2124 |
| **ALT (IU/L)** | 0.2732 | 0.4714 | 0.641 | 0.5791 | 0.0695 | 0.1182 | 0.0991 | 0.7992 | 0.8689 | 0.929 |
| **Smoking status** | 0.5213 | 0.6676 | 0.1114 | 0.9538 | 0.6315 | 0.2193 | 0.3815 | 0.488 | 0.6536 | 0.1151 |
| **Alcohol intake** | 0.1298 | 0.0049 | <.0001 | <.0001 | 0.298 | 0.0016 | 0.0004 | 0.1003 | 0.0318 | 0.8178 |
| **Regular exercise** | <.0001 | <.0001 | <.0001 | <.0001 | 0.634 | 0.0032 | 0.0003 | 0.0136 | 0.0018 | 0.5086 |
| **Hypertension** | 0.39 | 0.4652 | 0.4905 | 0.1119 | 0.8973 | 0.8649 | 0.4647 | 0.9673 | 0.3899 | 0.3675 |
| **Diabetes mellitus** | 0.9037 | 0.6871 | 0.0596 | 0.2184 | 0.6004 | 0.045 | 0.1764 | 0.1383 | 0.4074 | 0.5128 |
| **Dyslipidemia** | 0.1434 | 0.7084 | 0.0846 | 0.0046 | 0.2759 | 0.7938 | 0.1704 | 0.1768 | 0.014 | 0.2671 |
| **Residential area** | <.0001 | <.0001 | <.0001 | <.0001 | 0.1866 | <.0001 | <.0001 | 0.0066 | <.0001 | 0.0003 |
| **Total energy intake (kcal/day)** | <.0001 | <.0001 | <.0001 | <.0001 | <.0001 | <.0001 | <.0001 | <.0001 | <.0001 | <.0001 |
| **Carbohydrate intake (g/day)** | <.0001 | <.0001 | <.0001 | <.0001 | <.0001 | <.0001 | <.0001 | <.0001 | <.0001 | <.0001 |
| **Carbohydrate (%)** | <.0001 | <.0001 | <.0001 | <.0001 | <.0001 | <.0001 | <.0001 | <.0001 | <.0001 | <.0001 |
| **Fat intake (g/day)** | <.0001 | <.0001 | <.0001 | <.0001 | <.0001 | <.0001 | <.0001 | <.0001 | <.0001 | <.0001 |
| **Fat (%)** | <.0001 | <.0001 | <.0001 | <.0001 | <.0001 | <.0001 | <.0001 | <.0001 | <.0001 | <.0001 |
| **Protein intake (g/day)** | <.0001 | <.0001 | <.0001 | <.0001 | <.0001 | <.0001 | <.0001 | <.0001 | <.0001 | <.0001 |
| **Protein (%)** | <.0001 | <.0001 | <.0001 | <.0001 | <.0001 | <.0001 | <.0001 | <.0001 | <.0001 | <.0001 |

BMI, body mass index; WC, waist circumference; SBP, systolic blood pressure; DBP, diastolic blood pressure; FBG, fasting blood glucose; TC, total cholesterol; HDL-C, high density lipoprotein cholesterol; LDL-C, low density lipoprotein cholesterol; TG, triglyceride; BUN, blood urea nitrogen; Cr, creatinine; AST, aspartate transaminase; ALT, alanine transaminase;

**Supplementary Table S3. Multiple Cox proportional hazard regression analysis for all-cause mortality of protein intake quintile according to presence of diabetes**

|  | Adjusted HR (95%CI) | P-value |  | Adjusted HR (95% CI) | P-value |
| --- | --- | --- | --- | --- | --- |
| Without diabetes |  |  | With diabetes |  |  |
| Protein intake (g/kg/day) |  |  | Protein intake (g/kg/day) |  |  |
| Q1 (0.179, 0.548) | Ref |  | Q1 (0.239, 0.533) | Ref |  |
| Q2 (0.548, 0.689) | 0.88 (0.66-1.19) | 0.409 | Q2 (0.533, 0.660) | 1.05 (0.60-1.82) | 0.873 |
| Q3 (0.688, 0.843) | 0.82 (0.59-1.13) | 0.223 | Q3 (0.660, 0.791) | 1.23 (0.66-2.29) | 0.516 |
| Q4 (0.843, 1.047) | 1.03 (0.72-1.48) | 0.872 | Q4 (0.792, 1.021) | 1.64 (0.84-3.18) | 0.145 |
| Q5 (1.047, 3.450) | 1.05 (0.66-1.67) | 0.848 | Q5 (1.022, 3.573) | 1.60 (0.68-3.79) | 0.285 |
| Protein intake (%) |  |  | Protein intake (%) |  |  |
| Q1 (6.93, 10.49) | Ref |  | Q1 (7.51, 10.65) | Ref |  |
| Q2 (10.50, 11.72) | 0.96 (0.73-1.25) | 0.738 | Q2 (10.65, 11.76) | 0.72 (0.42-1.23) | 0.228 |
| Q3 (11.72, 12.90) | 0.87 (0.65-1.15) | 0.312 | Q3 (11.77, 12.96) | 0.80 (0.47-1.34) | 0.393 |
| Q4 (12.90, 14.54) | 0.95 (0.71-1.26) | 0.719 | Q4 (12.97, 14.64) | 0.96 (0.57-1.61) | 0.866 |
| Q5 (14.54, 24.74) | 0.90 (0.66-1.22) | 0.482 | Q5 (14.67, 25.54) | 0.97 (0.58-1.64) | 0.921 |

Adjusted for age, SEX, BMI, smoking, alcohol intake, exercise, total calorie, hypertension, and dyslipidemia

**Supplementary Table 4. Multiple Cox proportional hazard regression analysis for all-cause mortality of protein intake quintile according to total calorie intake.**

| Total calorie intake <20kcal/kg | | |  |  |  |  |
| --- | --- | --- | --- | --- | --- | --- |
|  | Model 1 |  | Model 2 |  | Model 3 |  |
| Variables | HR (95% CI) | p-value | HR (95% CI) | p-value | HR (95% CI) | p-value |
| Protein intake (g/kg/day) | | | | | | |
| Q1 (0.179, 0.386) | Ref |  | Ref |  | Ref |  |
| Q2 (0.387, 0.461) | 0.63(0.39-1.02) | 0.058 | 0.82(0.48-1.42) | 0.482 | 0.81(0.47-1.40) | 0.449 |
| Q3 (0.461, 0.526) | 0.93(0.60-1.42) | 0.728 | 1.26(0.74-2.17) | 0.396 | 1.25(0.73-2.14) | 0.41 |
| Q4 (0.526, 0.608) | 0.61(0.36-1.02) | 0.059 | 0.87(0.47-1.61) | 0.652 | 0.84(0.45-1.56) | 0.581 |
| Q5 (0.608, 1.000) | 0.72(0.44-1.17) | 0.182 | 1.15(0.61-2.16) | 0.668 | 1.10(0.58-2.06) | 0.775 |
| Protein intake (g/kg/day) | | | | | | |
| <0.8 | Ref |  | Ref |  | Ref |  |
| 0.8-1.2 | 1.02(0.38-2.77) | 0.961 | 1.23(0.45-3.38) | 0.684 | 1.27(0.46-3.51) | 0.643 |
| >1.2 | n/a |  | n/a |  | n/a |  |
| Protein intake (%) | | | | | | |
| Q1 (7.4, 10.2) | Ref |  | Ref |  | Ref |  |
| Q2 (10.2, 11.4) | 1.06(0.68-1.65) | 0.799 | 1.15(0.73-1.80) | 0.555 | 1.07(0.68-1.69) | 0.761 |
| Q3 (11.4, 12.7) | 0.78(0.48-1.27) | 0.312 | 0.84(0.51-1.39) | 0.501 | 0.81(0.49-1.33) | 0.406 |
| Q4 (12.7, 14.3) | 1.01(0.62-1.64) | 0.965 | 1.07(0.65-1.76) | 0.782 | 1.02(0.62-1.68) | 0.93 |
| Q5 (14.3, 24.0) | 0.89(0.54-1.46) | 0.65 | 1.04(0.63-1.74) | 0.872 | 1.01(0.60-1.68) | 0.983 |
| Total calorie intake ≥20kcal/kg | |  |  |  |  |  |
|  | Model 1 |  | Model 2 |  | Model 3 |  |
| Variables | HR (95% CI) | p-value | HR (95% CI) | p-value | HR (95% CI) | p-value |
| Protein intake (g/kg/day) |  |  |  |  |  |  |
| Q1 (0.365, 0.660) | Ref |  | Ref |  | Ref |  |
| Q2 (0.660, 0.782) | 0.93(0.70-1.22) | 0.585 | 0.96(0.72-1.27) | 0.75 | 0.94(0.71-1.25) | 0.677 |
| Q3 (0.782, 0.920) | 0.81(0.60-1.08) | 0.153 | 0.91(0.67-1.24) | 0.554 | 0.90(0.66-1.22) | 0.484 |
| Q4 (0.920, 1.129) | 1.06(0.80-1.39) | 0.704 | 1.20(0.87-1.66) | 0.257 | 1.19(0.86-1.65) | 0.286 |
| Q5 (1.129, 3.573) | 0.79(0.58-1.08) | 0.141 | 1.05(0.68-1.62) | 0.839 | 1.01(0.65-1.56) | 0.971 |
| Protein intake (g/kg/day) |  |  |  |  |  |  |
| <0.8 | Ref |  | Ref |  | Ref |  |
| 0.8-1.2 | 1.01(0.83-1.24) | 0.902 | 1.12(0.89-1.41) | 0.327 | 1.11(0.88-1.39) | 0.386 |
| >1.2 | 0.78(0.57-1.08) | 0.133 | 1.01(0.65-1.56) | 0.974 | 0.97(0.63-1.50) | 0.892 |
| Protein intake (%) |  |  |  |  |  |  |
| Q1 (6.9, 10.6) | Ref |  | Ref |  | Ref |  |
| Q2 (10.6, 11.8) | 0.76(0.57-1.01) | 0.059 | 0.81(0.61-1.08) | 0.159 | 0.79(0.59-1.05) | 0.102 |
| Q3 (11.8, 13.0) | 0.78(0.59-1.04) | 0.089 | 0.87(0.65-1.15) | 0.323 | 0.85(0.64-1.13) | 0.27 |
| Q4 (13.0, 14.6) | 0.84(0.63-1.13) | 0.246 | 0.90(0.67-1.22) | 0.501 | 0.89(0.66-1.20) | 0.445 |
| Q5 (14.6, 25.5) | 0.79(0.59-1.07) | 0.127 | 0.88(0.65-1.20) | 0.431 | 0.86(0.63-1.17) | 0.325 |

Model 1: adjusted for age, SEX, and BMI

Model 2: adjusted for age, SEX, BMI, smoking, alcohol intake, exercise, and total calorie

Model 3: adjusted for age, SEX, BMI, smoking, alcohol intake, exercise, total calorie, hypertension, diabetes, and dyslipidemia
